# Dominant female meerkats do not use aggression to elevate work rates of helpers in response to increased brood demand

**DOI:** 10.1016/j.anbehav.2011.12.032

**Published:** 2012-03

**Authors:** Peter Santema, Tim Clutton-Brock

**Affiliations:** Department of Zoology, University of Cambridge, Cambridge, U.K.

**Keywords:** coercion, cooperative breeding, meerkat, pay to stay, punishment, *Suricata suricatta*

## Abstract

In cooperatively breeding animals, in which nonbreeding subordinates assist in rearing offspring born to dominants, breeders and helpers may be in conflict over their respective contributions to offspring care and selection may favour breeders that use aggression to elevate the work rates of helpers. We tested the prediction that dominant female meerkats, *Suricata suricatta*, should increase aggression towards subordinates when the need for help is higher, by playing back recordings of pup begging calls to simulate increased need for help. Second, we tested the prediction that dominants should reduce aggression when subordinates help more, by playing back recordings of feeding calls to simulate elevated pup provisioning rates by subordinates. Neither of the two playback experiments affected rates of aggressive interactions between breeding females and helpers. Instead, breeding females increased their own level of pup provisioning in response to increased pup begging. Hence, our results do not support a role of aggression in regulating helping behaviour in meerkats, but suggest that pup provisioning can be explained by direct and/or indirect benefits derived from helping. As yet, firm evidence that breeders use aggression to promote helping by subordinates in cooperative animal societies remains elusive.

## Highlights

► In cooperative breeders, dominants may use aggression to elevate work rates of subordinates. ► Increased brood demand and helping effort were simulated in meerkats using playback experiments. ► Dominant females did not adjust aggressiveness towards helpers. ► Dominant females adjusted their own pup provisioning rates. ► Alloparental care by meerkat helpers is not enforced by dominants.

In cooperatively breeding animals, in which nonbreeding subordinates assist in rearing offspring born to dominants, there may be conflicts of interest between breeders and helpers over their respective contributions to offspring care (Reeve 1992). Such breeder–helper conflict over work rates may be expected whenever (1) breeder fitness is improved by helper contributions to offspring care (Emlen & Wrege 1991; Russell et al. 2007; Kingma et al. 2010), and (2) alloparental care is costly for helpers to provide (Rabenold 1990; Heinsohn & Legge 1999; Russell et al. 2003a). Under these conditions, selection may favour breeders that use aggression to elevate the work rates of helpers. Aggression may lead to elevated helper contributions to offspring care in two ways. First, aggressive interactions may be costly to helpers (Clutton-Brock & Parker 1995) and helpers need to elevate their work rates to avoid these costs. Alternatively, aggressive interactions may not be costly in themselves, but may function as a threat of punishment, such as physical attack or eviction from the group (Cant & Johnstone 2009; Cant 2011), and helpers need to elevate their work rates to avoid triggering such threats.

If dominants use aggression to promote helping by subordinates, it is expected that (1) aggression between dominants and subordinates should decline with the level of help provided by subordinates (Gaston 1978; Kokko et al. 2002; Wong & Balshine 2011) and (2) dominants should increase aggressiveness towards subordinates when the need for help is higher (Mulder & Langmore 1993; Wong & Balshine 2011). In a study on superb fairy-wrens, *Malurus cyaneus*, male helpers were prevented from helping by temporary removal from the group; upon return to the group, they were frequently chased by the dominant male and sometimes subjected to physical attack (Mulder & Langmore 1993), suggesting that helpers were subjected to aggression for their defection from helping. Moreover, helpers were subjected to increased aggression from the dominant male when the removal had happened during the incubation or chick-provisioning period, but not when the removal was done during the nonbreeding period, suggesting that helpers were subjected to increased aggression only when the need for help was high (Mulder & Langmore 1993). In a study on the Lake Tanganyika cichlid *Neolamprologus pulcher*, helpers were attacked by other helpers and sometimes evicted from the group after having been prevented from helping by temporary removal from the group, also suggesting that helpers were subjected to increased aggression for their temporary defection from helping (Balshine-Earn et al. 1998). Furthermore, in naked mole-rats, *Heterocephalus glaber*, helpers that contribute less to cooperative activities receive more aggressive interactions from the dominant female than industrious helpers (Reeve 1992), suggesting that breeders use aggression to elevate work rates of helpers.

These studies have been widely cited as support for the notion that dominants use aggression to elevate helping behaviour by subordinates. The interpretation of the results, however, has subsequently been questioned (Young et al. 2005; Wong & Balshine 2011). As temporary removal from the group is likely to affect dominance relations, increased rates of aggression after temporary removal may also be explained by intensified conflict over rank, which may be more pronounced during the breeding season. Moreover, in cichlids, temporarily removed helpers were only attacked by other helpers of the same size and sex (and not by breeders; Balshine-Earn et al. 1998), suggesting that conflict over rank may be a more plausible explanation for the increase in aggression (Young et al. 2005; Wong & Balshine 2011). In naked mole-rats, subsequent work showed that individuals that contribute little to cooperative activities are also those that are most likely to attempt to reproduce and that increased aggression towards lazy helpers may therefore be more likely to reflect conflict over reproduction (Jacobs & Jarvis 1996). As yet, conclusive evidence that breeders in cooperative animal societies use aggression to elevate work rates of their helpers remains elusive, and more direct tests are needed.

In this study, we experimentally tested in the cooperatively breeding meerkat, *Suricata suricatta*, (1) whether breeding females increase aggression towards helpers when the demand for help is higher and (2) whether breeding females reduce aggression towards helpers when the level of help provided is higher. Meerkats live in groups of up to 50 individuals with a dominant breeding pair that largely monopolizes reproduction and a number of related and unrelated helpers of both sexes (Griffin et al. 2003). Litters of up to six pups are produced two to four times per year and raised cooperatively by the group (Clutton-Brock et al. 1999a). Adults feed invertebrate and small vertebrate prey to the pups from the time they start accompanying the group at about 30 days up to nutritional independence at about 90 days (Brotherton et al. 2001; Clutton-Brock et al. 2002). During the period of dependence, pups continuously produce begging calls when running between group members to solicit food (Manser & Avey 2000). When a potential feeder locates a food item, pups change from the normal begging calls to a distinct high-pitched vocalization in anticipation of being fed (Manser & Avey 2000), here referred to as a ‘feeding call’.

Dominant females are likely to benefit from elevating the work rates of subordinates. Helpers have positive effects on the growth and survival of weaned pups (Clutton-Brock et al. 2001a; Russell et al. 2003b), and allow dominant females to reduce investment in raising litters (Clutton-Brock et al. 1998a, 2001a). Moreover, helper contributions positively affect the likelihood of those pups attaining direct reproduction during adulthood (Russell et al. 2007). Contributions to pup rearing are also costly for helpers, at least over the short term, as helpers that contribute more to pup rearing during a breeding attempt suffer substantial weight loss (Clutton-Brock et al. 1998a; Russell et al. 2003a). Because of differences that male helpers and female helpers face in the trade-off between helping effort and future reproduction, conflict over work rate with the dominant female is likely to be different for male and female helpers (Reeve 1992). As fecundity is tightly linked to females’ body condition (Russell et al. 2003b), contributions to alloparental care are likely to be traded off against potential future reproduction. Hence, work rate conflict is likely to be more acute between breeders and female helpers than between breeders and male helpers.

In this study, we tested whether dominant females increase rates of aggression towards subordinates in response to increased brood demand, which was simulated by playing back pup begging calls. Second, we tested whether dominant females reduce rates of aggression in response to higher levels of pup feeding by helpers, which was simulated by playing back feeding calls. In addition, we tested whether the playbacks differentially affected aggression rates of dominant females towards male and female helpers and whether dominant females adjusted their own rates of pup provisioning in response to the playbacks.

## Methods

### Study Site and Population

This study was conducted in the Kalahari desert, South Africa (26°58′S, 21°49′E), between November 2010 and May 2011, with permission of the Northern Cape Conservation Authority. The study site consists of the dry riverbeds of the Kuruman River, herbaceous flats and vegetated dunes. The ecological conditions and climate of the study site have been described elsewhere (Clutton-Brock et al. 1999b; Russell et al. 2002). One meerkat in each group is fitted with a radiocollar as part of the ongoing research, thereby allowing groups to be easily located (Scantlebury et al. 2002). All individuals are habituated to close observation (from < 1 m) and can be identified by a unique pattern of dye marks on their fur, which is maintained while individuals are resting without the need to disturb or capture them. Groups are visited at least once every 3 days and the age of almost all individuals is therefore known to within a few days. Dominance status of females can be readily identified, as the dominant female is the primary breeder in the group (Clutton-Brock et al. 2001b; Griffin et al. 2003) while all subordinate females are behaviourally submissive to her (Clutton-Brock et al. 1998b; O’Riain et al. 2000). Following previous studies, individuals are referred to as pups until they reach 90 days of age and are able to forage independently (Brotherton et al. 2001). Individuals are referred to as adults if they are older than 1 year and have the potential to breed (Brotherton et al. 2001).

### Recording and Playback Protocol

For the increased brood demand experiment, 36 recordings of continuous begging calls, each of 10 min, were taken from 18 different pups of six different groups. Feeding calls, which are produced when the pups are fed, were cut from the recordings. For the control, 12 recordings of background noise, each of 10 min, were taken at different locations across the study population. For the increased helping effort experiment, recordings of 36 feeding events were taken from 13 different pups of four different groups. These recordings were cut to include 10 s of begging calls followed by feeding calls (6 ± 2 s) to create playback stimuli of a begging pup receiving a food item. For the control, recordings of the same pups were cut to include 15 s of begging calls (and no feeding calls) to create playback stimuli of a begging pup not receiving a food item. All recordings were made using a Sennheiser ME66/K6 microphone connected to a Marantz PMD670 (WAV format, sample frequency 44.1 kHz, resolution 16 bit), during the peak pup provisioning period, when the pups were between 40 and 70 days of age.

All recordings were played back from an Archos MP3 player connected to a JBL loudspeaker at a volume adjusted to match the amplitude of natural calls. Treatment and control recordings were always presented on consecutive days at a group, and in a random order, during the peak provisioning period.

### Observation Protocol

Continuous focal behavioural data (Altmann 1974) were entered directly onto a handheld Psion organiser, programmed to act as a data logger that allows recording of behaviours to the nearest second. During focal observations of dominant females, all aggressive interactions with adult subordinates were recorded together with the identity of the participants. In addition, the proportion of time spent within 1 m of an adult subordinate, the number of food items that were found and the number of food items fed to a pup were recorded. An aggressive interaction was said to have occurred if the dominant female subjected a subordinate to one of the following behaviours: growling (the dominant female makes growling vocalizations upon encountering a subordinate), hip-slam (the dominant female attempts to displace a subordinate with a sideways motion of the hips), chin-mark (the dominant female rubs its chin on a subordinate) or foraging competition (the dominant female aggressively attempts to take a food item away from the subordinate; Young 2003; Kutsukake & Clutton-Brock 2006). Playback and observation were paused if foraging was interrupted for more than 1 min (e.g. by alarm calls or interactions with neighbouring groups) and continued when foraging had been resumed by more than half the group.

### Increased Brood Demand Experiment

To simulate increased brood demand, 20 min playback stimuli were created by merging two randomly selected 10 min recordings of pup begging calls. Four different 20 min recordings were then played back, each separated by 5 min, at a distance of approximately 3 m from the dominant female. As pups normally beg in close proximity (<2 m) to the dominant female for an average of 19.7% of the time (P. Santema unpublished data), the treatment represents a significant increase in exposure to pup begging. New playback stimuli were created for each trial, ensuring that no two groups received the same set of playback stimuli. To monitor the behaviour of the dominant female, focal observations were performed simultaneously with the playbacks, totalling 80 min of observation per treatment. Adults responded to the speakers in the same way as they respond to real pups, and repeatedly brought food items to the speaker. In these instances, the playback was briefly paused after which the food item was either delivered to the nearest pup (61%) or eaten by the adult itself (39%). To habituate the meerkats to the playbacks, a randomly selected 10 min recording of begging calls was played back prior to the treatment, during which the volume was slowly increased to natural begging volume. No data were recorded during this habituation playback and treatment started 5 min after the habituation period.

For the control, 20 min playback stimuli were created by merging two randomly selected 10 min recordings of background noise. Four different 20 min recordings were then played back, separated by 5 min, at approximately 3 m from the dominant female. Focal observations of the dominant female were performed simultaneously with the playbacks, totalling 80 min of observation per treatment. To habituate the meerkats to the playback, a randomly selected 10 min recording of background noise was played back prior to the treatment, during which no data were recorded. Control playback and data collection started 5 min after this habituation period. A total of 10 trials (treatment plus control) were performed, one for the dominant female in each of 10 different groups, totalling 1600 observation minutes.

### Increased Helping Effort Experiment

To simulate increased pup provisioning by helpers, playback stimuli were created by merging five randomly selected recordings of feeding events (begging calls followed by feeding calls), each separated by 1 min. Four different playback stimuli of five feeding events were then played back at approximately 3 m from the dominant female, each followed by 20 min of focal behavioural observation of the dominant female. This resulted in a total number of 20 feeding event playbacks and a total observation period of 80 min. New playback stimuli were created for each trial, ensuring that no two groups received the same set of playback stimuli. As natural pup feeding rates during the peak provisioning period average 20 pup feeds/h (unpublished data), the treatment represents a significant increase in feeding calls.

For the control, playback stimuli were created by merging five randomly selected recordings of begging calls (not followed by feeding calls), each separated by 1 min. Four different playback stimuli of five begging recordings were then played back at approximately 3 m from the dominant female, each followed by 20 min of focal behavioural observation of the dominant female, totalling 80 min of observation. New playback stimuli were created for each trial, ensuring that no two groups received the same set of playback stimuli. A total of 12 trials (treatment plus control) were performed, one for the dominant female in each of 12 different groups, totalling 1920 observation minutes.

## Results

### Increased Brood Demand Experiment

**Figure 1:**
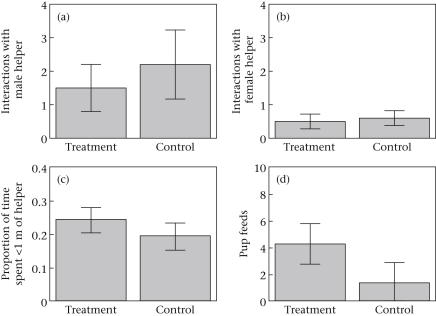
Increased brood demand experiment. The effect of simulated increased brood demand on (a) the number of aggressive interactions between the dominant female and male helpers, (b) the number of aggressive interactions between the dominant female and female helpers, (c) the proportion of time the dominant female spent within 1 m of a helper and (d) the number of pup feeds by the dominant female. Figures represent mean per 80 min observation period ± SE.

A total of 49 aggressive interactions between the dominant female and an adult subordinate were observed (*N* = 10 trials, 1600 observation minutes). There was no difference between the treatment and control playbacks in either the total number of aggressive interactions with subordinates (Wilcoxon signed-ranks test: *V* = 25.5, *N* = 10, *P* = 0.32; treatment: mean ± SE = 2.0 ± 0.67 per observation; control: mean ± SE = 2.9 ± 1.24 per observation) or the number of aggressive interactions with subordinate males (Wilcoxon signed-ranks test: *V* = 17, *N* = 10, *P* = 0.20; Fig. 1a) or subordinate females (Wilcoxon signed-ranks test: *V* = 8.5, *N* = 10, *P* = 0.89; Fig. 1b) when analysed for each sex separately. There was also no difference between the treatment and control playbacks in the proportion of time spent in close proximity (<1 m) to an adult subordinate (Wilcoxon signed-ranks test: *V* = 13.5, *N* = 10, *P* = 0.17; Fig. 1c).

A total of 57 pup feeds by dominant females were observed. Dominant females fed pups more often in the treatment than in the control, both when measured as the total number of food items fed to pups (Wilcoxon signed-ranks test: *V* = 2.5, *N* = 10, *P* = 0.033; Fig. 1d) and as the proportion of food items fed out of the number of food items found (Wilcoxon signed-ranks test: *V* = 3.5, *N* = 10, *P* = 0.050; treatment: mean ± SE = 0.17 ± 0.06; control: mean ± SE = 0.06 ± 0.02).

### Increased Helping Effort Experiment

**Figure 2:**
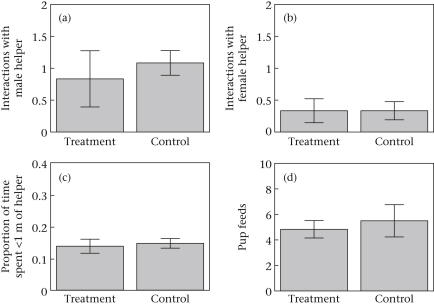
Increased helping effort experiment. The effect of simulated increase in helping effort by subordinates on (a) the number of aggressive interactions between the dominant female and male helpers, (b) the number of aggressive interactions between the dominant female and female helpers, (c) the proportion of time the dominant female spent within 1 m of a helper and (d) the number of pup feeds by the dominant female. Figures represent mean per 80 min observation period ± SE.

A total of 31 aggressive interactions between the dominant female and an adult subordinate were observed (*N* = 13 trials, 1920 observation minutes). There was no difference between the treatment and control playbacks in either the total number of aggressive interactions with subordinates (Wilcoxon signed-ranks test: *V* = 27, *N* = 12, *P* = 0.63; treatment: mean ± SE = 1.17 ± 0.47 per observation; control: mean ± SE = 1.42 ± 0.19 per observation) or the number of aggressive interactions towards subordinate males (Wilcoxon signed-ranks test: *V* = 41.5, *N* = 12, *P* = 0.46; Fig. 2a) or subordinate females (Wilcoxon signed-ranks test: *V* = 7.5, *N* = 12, *P* = 1; Fig. 2b) when analysed for each sex separately. There was also no difference between the treatment and control playbacks in the proportion of time dominant females spent in close proximity (<1 m) to an adult subordinate (Wilcoxon signed-ranks test: *V* = 60, *N* = 12, *P* = 0.11; Fig. 2c).

A total of 124 pup feeds by the dominant female were observed. Pup feeding rates did not differ between treatment and control playbacks, whether measured as the total number of food items fed to pups (Wilcoxon signed-ranks test: *V* = 35, *N* = 12, *P* = 0.89; Fig. 2d) or as the proportion of food items fed out of the number of food items found (Wilcoxon signed-ranks test: *V* = 46, *N* = 12, *P* = 0.62; treatment: mean ± SE = 0.20 ± 0.03; control: mean ± SE = 0.23 ± 0.04).

## Discussion

We found no evidence that dominant female meerkats use aggression to elevate work rates of subordinates. First, dominant females did not increase rates of aggressive interactions towards either male or female helpers, nor did they spend more time in close proximity to helpers, when an increase in brood demand was simulated. Second, dominant females did not reduce rates of aggressive interactions towards either male or female helpers, nor did they spend less time in close proximity to helpers, when an increase in rates of pup provisioning by helpers was simulated.

We expected an increase in rates of aggression between dominant females and subordinates in response to playbacks of pup begging calls. One potential explanation for why this was not observed may be that the playbacks were not perceived by dominant females as an increase in brood demand (MacGregor & Cockburn 2002). This explanation seems unlikely, however, because adult breeders and helpers responded to speakers in the same way that they responded to real pups, repeatedly bringing food items to the speakers (see also English et al. 2008; Madden et al. 2009). It is also possible that dominants subject subordinates to aggression only under specific conditions, for instance when a subordinate finds a food item close to the dominant. Although we did not specifically test for this, the frequency of such events should be similar during the treatment and control playbacks and any effects of pup begging on the likelihood of aggression in such events should have been reflected in our data. Hence, our results suggest that dominant females respond to increased brood demand not by increasing aggression towards helpers, but by increasing their own levels of pup provisioning.

It was previously found that subordinate meerkats increase their rates of pup provisioning in response to experimental playback of begging calls (Carlson et al. 2006; English et al. 2008), but whether this was mediated by increased rates of aggression from the dominant female was not investigated. The results of this study show that this is unlikely to be the case, and that helpers most likely increase levels of pup provisioning in response to increased brood demand of their own accord. In addition, our results show that dominant females also increased their own levels of pup provisioning in response to increased begging. Hence, these results suggest that both subordinates and dominants adjust their own rates of pup provisioning to maximize their inclusive fitness through direct and/or indirect benefits derived from such helping behaviour. This reinforces previous explanations for the evolution of alloparental care in meerkats, that helpers gain a combination of direct fitness benefits through group augmentation and indirect fitness benefits from rearing close kin (Clutton-Brock et al. 2001a, 2002).

We expected a decrease in rates of aggressive interactions between the dominant female and subordinates in response to feeding call playbacks. As subordinates often feed pups out of sight from the dominant female, one of the few cues available to the dominant female to monitor provisioning rates by subordinates is the number of feeding calls produced by the pups. Dominant females, however, did not reduce rates of aggressive interactions towards subordinates in response to feeding call playbacks. Furthermore, as dominant females increased their levels of food provisioning in response to increased pup begging, a reduction in food provisioning in response to feeding call playbacks might have been expected in response to increased feeding calls, but this was not the case. One possibility is that dominants do not use feeding calls to assess the provisioning effort of helpers and that the playbacks were therefore not perceived by dominant females as an increased helping effort by subordinates. For instance, dominant females may indirectly assess helping effort by monitoring pup begging intensity, which remained unchanged in the experiment. Nevertheless, these results suggest that dominant females do not reduce rates of aggression in response to an increase in rates of feeding calls given a certain level of pup begging. Manipulating actual provisioning rates by subordinates (e.g. Wright & Dingemanse 1999), however, may provide a better means to test whether aggression from the dominant female changes according to the level of help provided.

Aggressive interactions are commonly observed in meerkats, especially from the dominant female directed towards subordinates, but the function of such aggression remains unclear. Theory suggests that aggression may function to resolve within-group conflict if aggressive interactions are costly to helpers (Clutton-Brock & Parker 1995) or if aggression functions as a threat of punishment (Cant & Johnstone 2009; Cant 2011), such that helpers need to modify their behaviour to avoid these costs. Our results show that rates of aggression did not change when conflict over work rate was manipulated, either through increased brood demand or increased helper contributions to offspring care. Hence, aggression does not seem to play a role in the context of conflict over contributions to offspring care in meerkats. Aggression in meerkats, however, is commonly observed in the context of conflict over reproduction (Kutsukake & Clutton-Brock 2006), suggesting that aggression might play a role in mitigating conflict over reproduction (P. Santema, unpublished data).

As yet, conclusive evidence that dominants use aggression to elevate work rates of subordinates in cooperative breeders remains elusive (see Introduction), despite the fact that conflicts over work rate are expected to arise in many cooperative breeders. Helper contributions to offspring care often have a positive effect on breeders’ fitness (Russell et al. 2007; Kingma et al. 2010), and often are costly for the helpers (Heinsohn & Legge 1999; Russell et al. 2003a). Why, then, do breeders not use aggression to elevate helping effort of subordinates? We suggest two factors that may contribute to the absence of a role for aggression in the context of conflict over offspring care. First, the benefits of using aggression may be limited and mechanisms to enforce helping effort may not be selected for. As limitations in reproductive opportunities prevent the possibility for direct reproduction by subordinates in many cooperative breeders (Hager & Jones 2009) and the only way for most helpers to enhance their fitness is by assisting in rearing the breeder’s offspring, conflict over helping effort between breeders and helpers may be limited. The use of aggression may also be potentially costly to dominant breeders. For instance, using aggression to increase helpers’ work rates may cause helpers to leave the group or challenge the dominant’s position (Buston & Zink 2009; Johnstone & Cant 2009). Furthermore, as helpers are often related to breeders (Griffin & West 2003), forcing helpers to provide costly help may reduce their chances of future reproduction and, ultimately, the dominant’s indirect fitness. Second, dominant breeders may often be unable to assess work rates of subordinates. The level of offspring care may not easily be assessed by breeders, as it requires accumulation of information of an individual’s work rates over a prolonged period of time. This may be particularly relevant in species such as meerkats in which adults forage in loosely dispersed groups (Turbé 2006), such that helpers are often out of sight from the breeders.

In summary, we did not find any evidence that dominant female meerkats use aggression to elevate the helping effort of subordinates. Indeed, among cooperative breeders, there is little conclusive evidence to support the notion that dominants use aggression to elevate the work rates of helpers, despite the fact that the potential for conflict over work rates between breeders and helpers is likely to be common. We believe that the benefits to breeders of enforcing helping may often be limited and that breeders may commonly be unable to keep track of helper contributions to offspring care. Therefore, attempts to investigate a role of aggression in promoting helping behaviour may best be targeted on those cooperative breeders in which the benefits of enforcing helping behaviour in subordinates are likely to be large (i.e. helping is costly, helping increases fitness of breeders, dominance asymmetries are large and relatedness between helpers and breeders is low) and breeders have the potential to monitor helping effort by subordinates (i.e. groups comprising small stable units in which offspring care takes place at a centralized site).
